# Reinvestigation of the Late Devonian Lycopsid *Sublepidodendron grabaui* from Anhui Province, South China

**DOI:** 10.3390/biology11101544

**Published:** 2022-10-21

**Authors:** Peng Xu, Le Liu, Deming Wang

**Affiliations:** 1 Key Laboratory of Orogenic Belts and Crustal Evolution, School of Earth and Space Sciences, Peking University, Beijing 100871, China; 2 School of Geoscience and Surveying Engineering, China University of Mining and Technology Beijing, Beijing 100083, China

**Keywords:** *Sublepidodendron grabaui*, Late Devonian, lycopsid, megasporangiate strobili, *Lagenicula*

## Abstract

**Simple Summary:**

The lycopsid *Sublepidodendron* is cosmopolitan in the Late Devonian and Early Carboniferous. *Sublepidodendron grabaui* is known from several localities of the Upper Devonian Wutong Formation in South China, with its overall morphology and male reproductive organs (i.e., the microsporangiate strobili and microspores) studied previously. Here we describe new specimens of *S. grabaui* from Guangde City, Anhui Province, to get further knowledge on this plant, especially its female reproductive organs (i.e., megasporangiate strobili and megaspores). The megasporangiate strobili are borne terminally on fertile axes and occasionally dichotomized, bearing at least eight *Lagenicula*-type megaspores with a small gula in each sporangium. Based on the previous and present study, this plant is considered as a tree lycopsid exhibiting multiple dichotomized stems, occasionally produced lateral branches and monosporangiate strobili.

**Abstract:**

South China displays Devonian strata with well-exposed outcrops and is regarded as a diversity hotspot of Late Devonian lycopsids. The heterosporous lycopsid *Sublepidodendron grabaui* has been studied for over ten years, with its general morphology, aerial stem anatomy, microsporangiate strobili, and growth architecture reported. Based on new specimens from Guangde City, Anhui Province, this study provides further knowledge about the megasporangiate strobili and megaspores of *S. grabaui*. Its slender megasporangiate strobili occur singly or in pairs and occasionally bifurcate in the middle-upper portion. Each megasporophyll consists of a flattened pedicel and an adaxially curved lamina. The lamina forms a downturned heel at the base. Each sessile megasporangium contains at least eight *Lagenicula*-type megaspores with a small gula. The other observed characteristics of *S. grabaui* in this study conform to those previously known and are compared to relative coeval taxa.

## 1. Introduction

The lycopsids dominated the Paleozoic wetland landscape and possessed a long evolutionary history, evidenced by the earliest fossil traced back to the late Silurian [1,2,3]. Middle Devonian lycopsids evolved many key characteristics, e.g., the heterospory [4,5] and the tree habit [6,7]. Heterosporous lycopsids blossomed in the Late Devonian (e.g., [8,9,10,11,12,13,14,15,16]), which is regarded as a major step in the full colonization of land surfaces by plants [17,18]. In the past 20 years, over ten species of Late Devonian heterosporous lycopsids have been reported or restudied all around the world (e.g., [9,10,11,12,13,14,15,16,19,20,21,22]); especially, the South China Plate held eight species with monosporangiate strobili (e.g., [9,10,11,12,13,14,15,16]), suggesting a probable origin center and diversity hotspot of this group.

*Sublepidodendron grabaui* is known as the combination of the former morphological species *S. wusihense* and *Lepidostrobus grabaui* and is described as an arborescent, heterosporous lycopsid from several localities of the Upper Devonian Wutong Formation in South China [12,23]. The previous studies revealed the general morphology, aerial stem anatomy, microsporangiate strobili, and growth architecture of *S. grabaui*. In this article, its megaporangiate strobili and megaspores are investigated based on new materials excavated from the Upper Devonian of Guangde City, Anhui Province, China.

## 2. Materials and Methods

**Figure 1:**
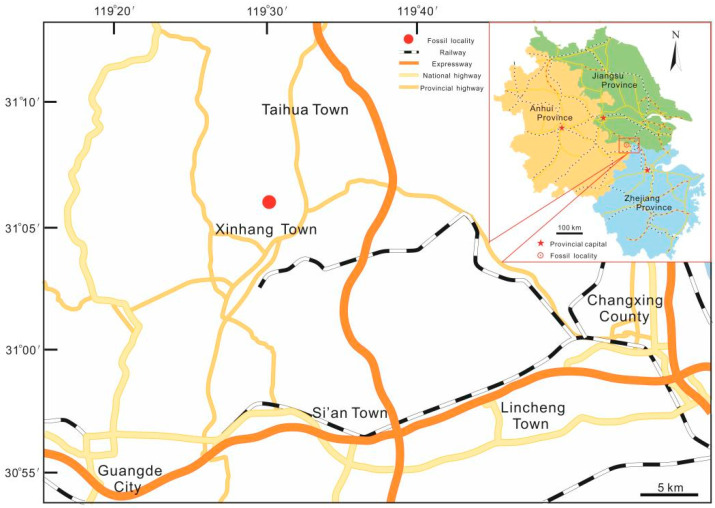
Map showing the fossil locality of *Sublepidodendron grabaui* in this study.

**Figure 2:**
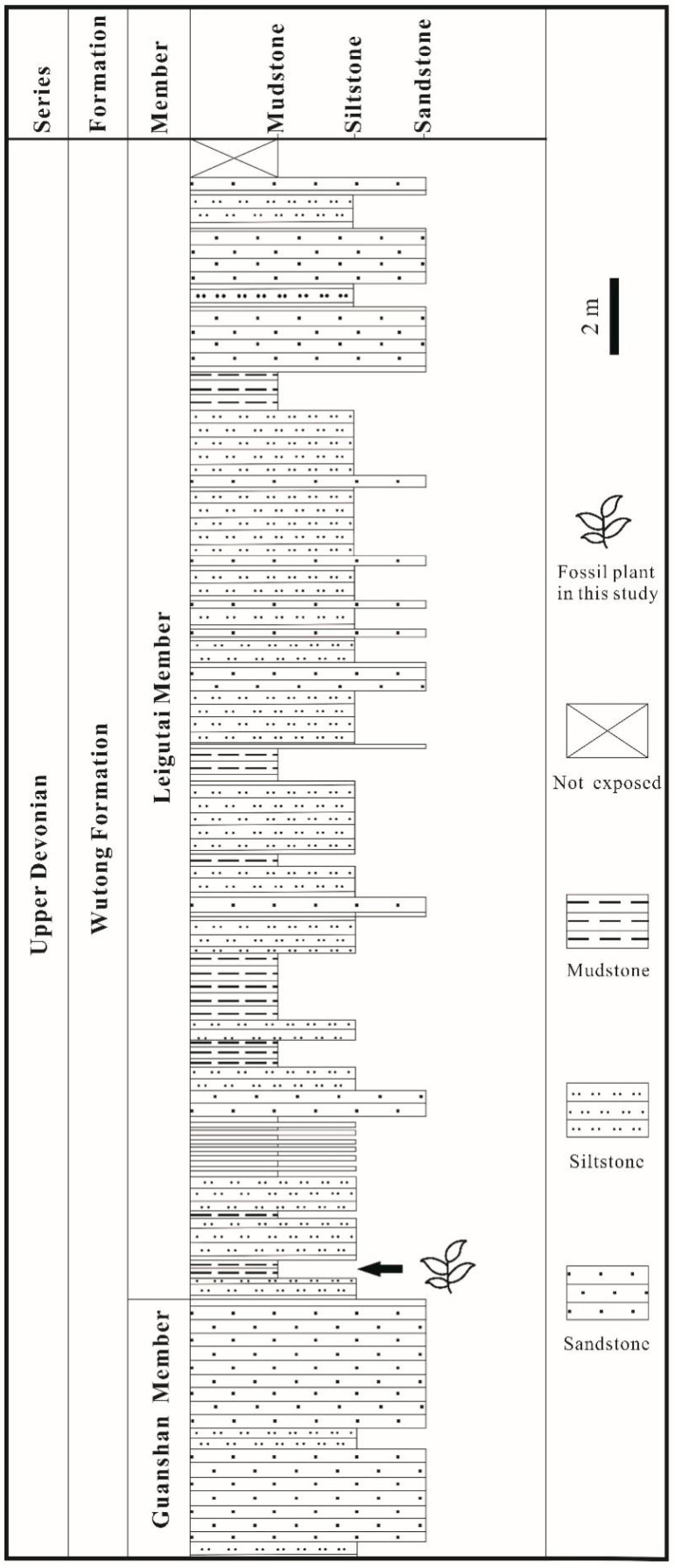
Stratigraphic column of the Yongchuan Section in Xinhang Town, Guangde City, Anhui Province, China, showing the fossil-bearing bed at the bottom part of Leigutai Member, Wutong Formation.

The specimens were collected from the Yongchuan mine, Xinhang town, Guangde City, Anhui Province, China (Figure 1). The fossil plant in this study is restricted to the bottom portion of the Leigutai Member, i.e., the upper member of the Upper Devonian (Famennian) Wutong Formation (Figure 2), and is preserved as compressions and impressions in yellow, brown, or grayish mudstones. Steel needles were used to expose the morphology of some dichotomized axes and strobili. The limonitized stele shows the anatomy in the transverse section, which was examined under a stereomicroscope. The specimens were photographed using a Nikon Z6 digital camera and a Leica S9 stereomicroscope. All figures were organized using Adobe Photoshop CC 2018 (ver. 19.0, San Jose, CA, USA) and Corel Draw 2018 (ver. 20.1, Ottawa, ON, Canada). All specimens are housed at the Department of Geology, Peking University, Beijing, China.

### Systematic Palaeobotany

Class: Lycopsida Pichi-Sermolli 1958.

Order: Isoëtales sensu lato Meyen 1987.

Suborder: Dichostrobiles DiMichele and Bateman 1996.

Family: Sublepidodendraceae Kräusel and Weyland 1949.

Genus: *Sublepidodendron* (Nathorst) Hirmer 1927.

Type species: *Sublepidodendron mirabile* (Nathorst) Hirmer 1927.

*Sublepidodendron grabaui* (Sze) Wang and Xu 2005 [23] emend. Meng et al., 2016 [12] emend.

Neotype: PB 2505 (first designated by Wang and Xu in Bot. J. Linn. Soc., 2005 [23], and deposited at NIGP-CAS).

Epitype: PB 19,255 (first designated by Wang and Xu in Bot. J. Linn. Soc., 2005 [23], and deposited at NIGP-CAS).

Specimens examined here: PKUB22000–22002, 22003a, 22003b, 22004–22009, 22010a, 22010b, 22011–22015 (Deposited at Department of Geology, Peking University, Beijing, China).

Emended specific diagnosis: (emended and additional generic characters are in brackets) Arborescent and heterosporous lycopsid plant with a trunk, branches, and monosporangiate strobili. Trunk, 32–100 mm wide. Branches, 1.5–24 mm wide terminate trunk, dichotomized at least four times or pseudomonopodially branched. Leaves, simple, linear, [inserted at 30–90° on the axis], 0.4–[2.0] mm wide at the proximal portion, 18–60 mm long, with a single midvein. Leaf bases, elongate fusiform in outline, 0.3–1.3 mm at the widest point, 1.6–20 mm long, with an obtusely rounded ridge at the widest point, and one vascular bundle and keel. Each branch terminated by a single cigar-shaped strobilus, [6.0–11] mm wide (excluding the distal part of sporophylls), up to 160 mm in total length. The stalk of sporophyll 0.2 mm wide and 3.0–4.0 mm long. Sporophyll laminae with smooth margins, rhomboid in outline, with pointed apex. Sporangia adaxial, elongate elliptical in outline, 0.8–[1.3] × [2.7]–4.0 mm, without obvious pedicels. [Megasporangiate strobili single, paired, or occasionally once-dichotomized. *Lagenicula* type] megaspores with circular amb, [a gula and spiny ornamentation, 661]–1200 μm in diameter. [Gula 289–440 μm high.] Cingulate *Lycospora*-type microspores, 18–35 μm in diameter, proximal surface with finely microgranulate ornamentation and distal surface with densely microspinate ornamentation. Trunk stele with a pith, exarch primary xylem, and secondary xylem; stele of large branches with a small pith, exarch primary xylem; a solid exarch primary xylem strand present in small branches.

## 3. Results

**Figure 3:**
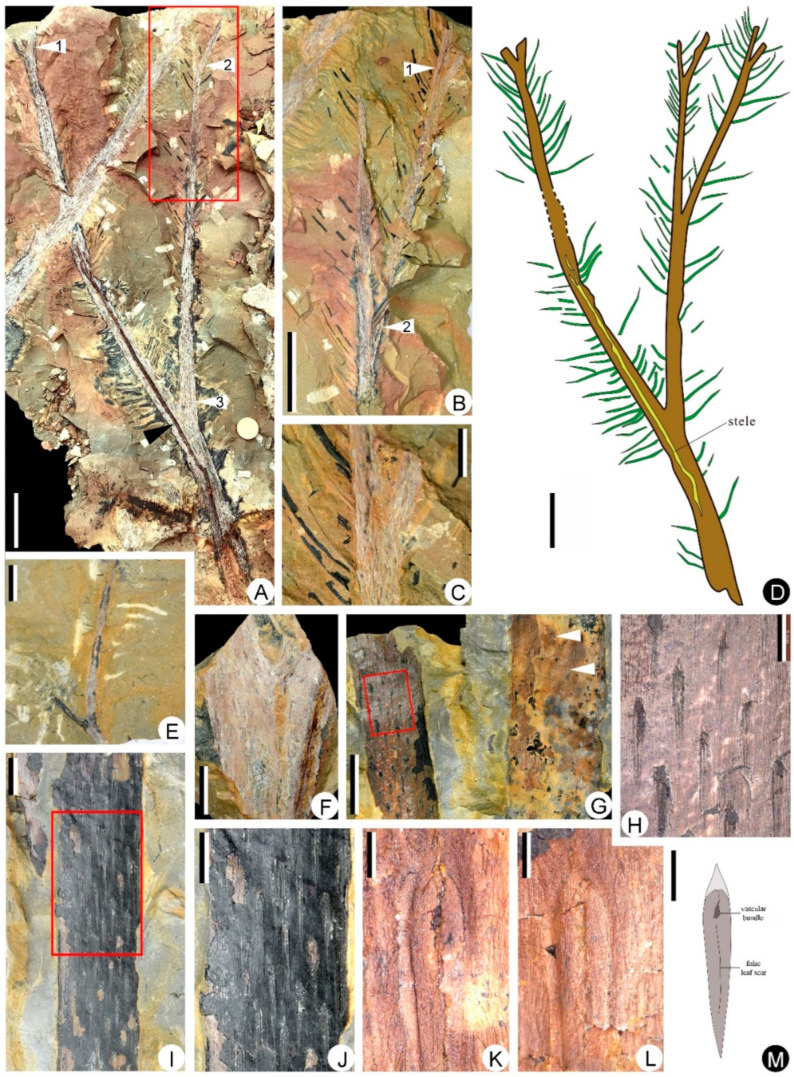
Stems and elongate-fusiform leaf bases of *Sublepidodendron grabaui*. (**A**) Stem dichotomizing several times. White arrows 1, 2, and 3 indicating the bifurcate portions. Rectangle indicating the counterpart enlarged in Figure 3B. The black arrow in the lower part indicating a limonitized stele in the stem. Scale bar = 5 cm. PKUB22010a. (**B**) Enlargement of the counterpart of Figure 3A (rectangle), showing stem dichotomizing two times. The arrow indicating the bifurcate portion. Scale bar = 5 cm. PKUB22010b. (**C**) Enlargement of Figure 3B (arrow 1), showing axes with a dichotomy. Scale bar = 2 cm. (**D**) Line illustration of the *Sublepidodendron grabaui* stems shown in Figure 3A–C. Stem with three times of dichotomy and dense leaves. The dotted lines indicating the missing portion of the stem. Scale bar = 5 cm. (**E**) A once-dichotomized slender axis. Scale bar = 2 cm. PKUB22008. (**F**) A once-dichotomized axis. Scale bar = 2 cm. PKUB22011. (**G**) Two stems with helically arranged leaf bases. The rectangle indicating the portion enlarged in Figure 3H. the arrows indicating the portions enlarged in Figure 3K (**upper**) and Figure 3L (**lower**), respectively. Scale bar = 2 cm. PKUB22006. (**H**) Enlargement of Figure 3G (rectangle), indicating elongate-fusiform leaf bases. Scale bar = 5 mm. (**I**) Stem with helically arranged leaf bases. The rectangle indicating the portion enlarged in Figure 3J. Scale bar = 1 cm. PKUB22014. (**J**) Enlargement of Figure 3I (rectangle), indicating elongate-fusiform leaf cushions. Scale bar = 5 mm. (**K**,**L**) Enlargement of Figure 3G (upper and lower arrows, respectively), showing vascular bundle scar and keel in leaf base. Scale bar = 2 mm. (**M**) Line illustration of a leaf base based on Figure 3H–L. Scale bar = 2 mm.

**Figure 4:**
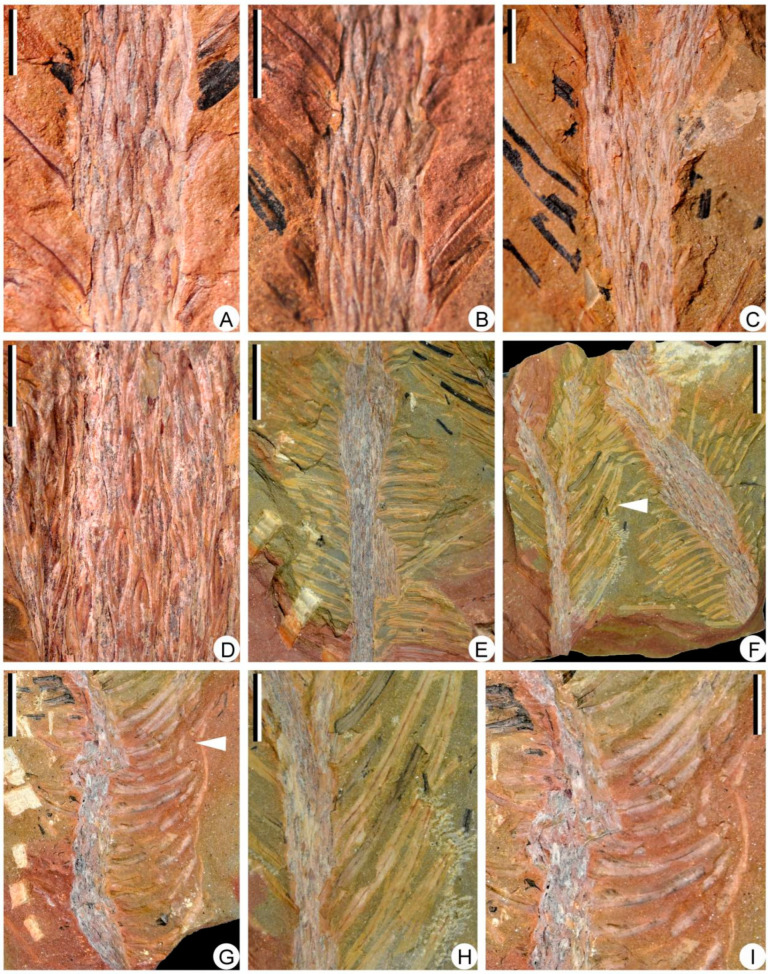
Leaf bases and vegetative leaves along slender axes of *Sublepidodendron grabaui*. (**A**–**C**) Different stems with helically arranged leaf bases. Scale bar = 5 mm. (**D**) Helically arranged leaf bases, each showing a vertical groove in the middle. Scale bar = 5 mm. (**E**) A vegetative axis with persistent dense leaves. Scale bar = 1 cm. (**F**) Two once-dichotomized vegetative axes with persistent linear leaves. The arrows indicating the portions enlarged in Figure 4H. Scale bar = 2 cm. PKUB22012. (**G**) A vegetative axis with persistent linear leaves. The arrows indicating the portions enlarged in Figure 4I. Scale bar = 2 cm. PKUB22001. (**H**,**I**) Enlargement of arrowed portion in Figure 4F and Figure 4G, respectively, showing vegetative leaves, each with a single midvein. Scale bar = 1 cm.

**Figure 5:**
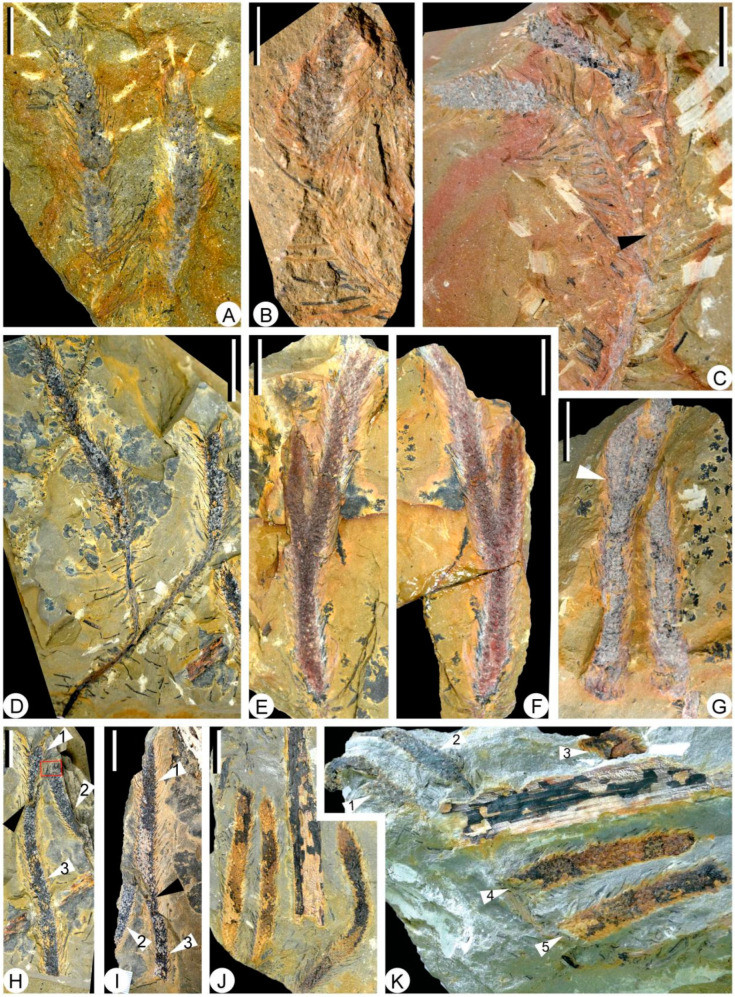
Fertile axes and strobili of *Sublepidodendron grabaui*. (**A**) Two strobili with slender sporophylls. Scale bar = 2 cm. PKUB22002. (**B**) A strobilus with basal fertile axis preserved. Scale bar = 2 cm. PKUB22004. (**C**) A once-dichotomized axis (arrow) bearing two strobili. Scale bar = 2 cm. PKUB22001. (**D**) A once-dichotomized axis bearing two strobili. Scale bar = 2 cm. PKUB22000. (**E**,**F**) Part and counterpart of a bifurcated strobilus. Scale bar = 1 cm. PKUB22003a, 22003b. (**G**) A once-dichotomized strobilus. The arrow indicating the bifurcate portion. Scale bar = 2 cm. PKUB22007. (**H**) Three broken slender strobili. Black arrow indicating the gap between two rock layers. Arrows 1, 2, and 3 indicating the three strobili, respectively. The rectangle indicating the portion enlarged in Figure 6A. Scale bar = 2 cm. (**I**) The counterpart of Figure 5H. Black arrow indicating the gap between two rock layers. Arrows 1, 2, and 3 indicating the three strobili shown in Figure 5H. Scale bar = 2 cm. (**J**) Three strobili preserved in the same direction. Scale bar = 2 cm. PKUB22015. (**K**) Five strobili (arrows 1–5) preserved in two different directions (arrows 1–3 and arrows 4 and 5, respectively). Scale bar = 1 cm. PKUB22005.

**Figure 6:**
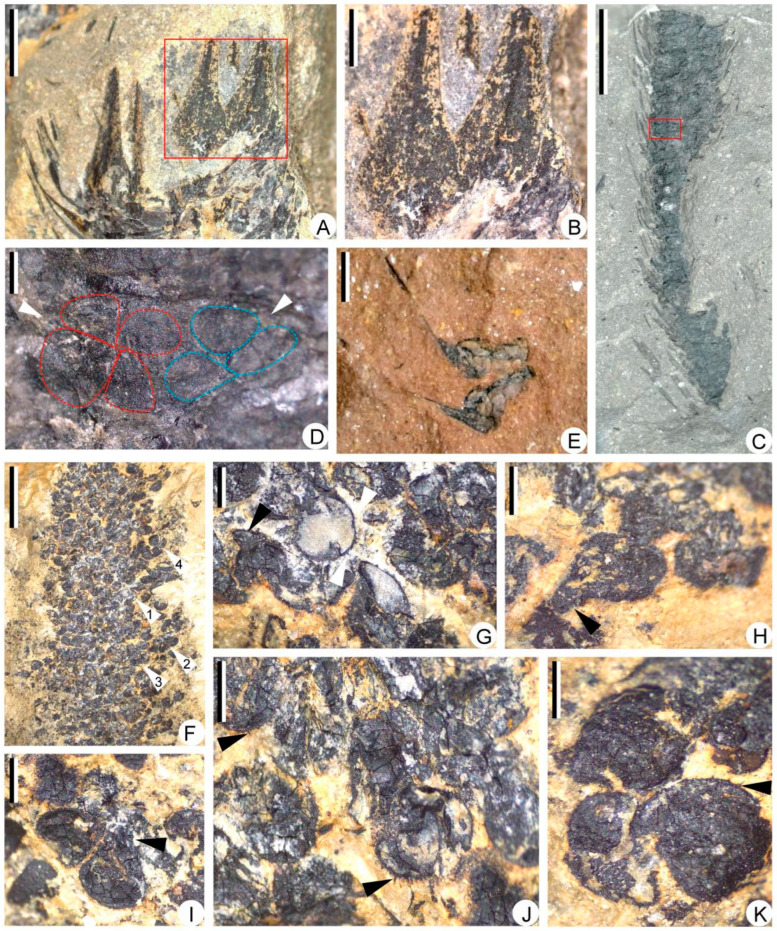
Megasporophylls and megaspores of *Sublepidodendron grabaui*. (**A**) Enlargement of portion in Figure 5H (rectangle), showing sporophylls in helices. Scale bar = 2 mm. (**B**) Enlargement of portion in Figure 6A (rectangle), showing front view of sporophyll laminae, each with a midvein. Scale bar = 1 mm. (**C**) A partially preserved strobilus. The rectangle indicating the portion enlarged in Figure 6D. Scale bar = 1 cm. PKUB22009. (**D**) Enlargement of Figure 6C (rectangle), showing contents of a sporangium. The arrows indicating two possible tetrads. The dotted line shows the outline of megaspores in tetrads. (**E**) Two isolated megasporophyll–sporangium complexes with lateral view of sporophylls. Scale bar = 2 mm. (**F**) Large numbers of megaspores. Arrows 1–4 indicating portions enlarged in Figure 6G,I–K, respectively. Scale bar = 5 mm. PKUB22013. (**G**) Enlargement of Figure 6F (arrow 1), showing megaspores with gula and spiny ornamentations. The black and white arrows indicating gula and spiny ornamentation, respectively. Scale bar = 500 μm. (**H**) A pear-shaped megaspore. The arrows indicating the position of the gula. Scale bar = 500 μm. (**I**) Enlargement of Figure 6F (arrow 2), showing two megaspores with gula. The arrow indicating the position of the gula. Scale bar = 500 μm. (**J**,**K**) Enlargement of Figure 6F (arrows 3 and 4). The arrow indicating the spiny ornamentations on the megaspore surface. Scale bar = 500 μm.

**Figure 7:**
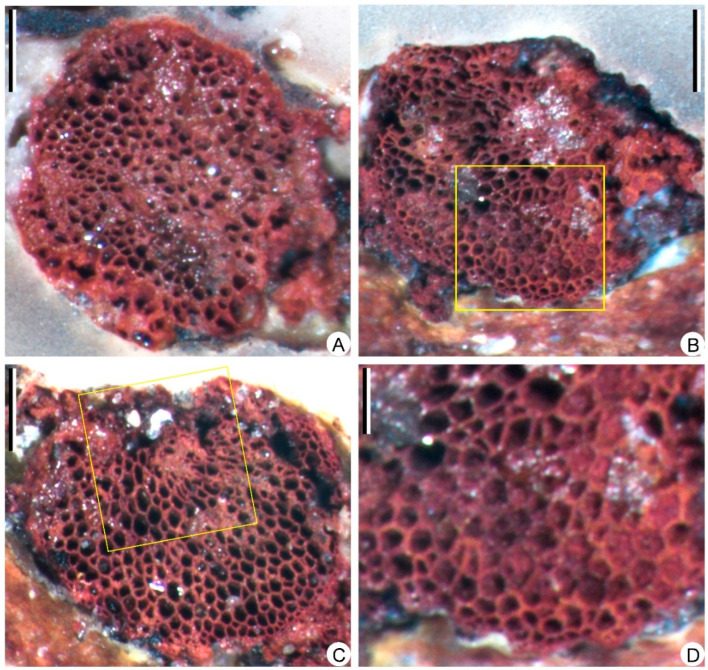
Transverse sections of the stele, showing the anatomy of *Sublepidodendron grabaui*. (**A**) Solid protostele. Scale bar = 1 mm. (**B**) Partially preserved stele. The rectangle indicating the portions enlarged in Figure 7D. Scale bar = 1 mm. (**C**) Partially preserved stele. The rectangle indicating the portions where tracheids are torn. Scale bar = 1 mm. (**D**) Enlargement of Figure 7B (rectangle) displaying the periphery of the stele with protoxylem tracheids. Scale bar = 400 μm.

The specimens of *Sublepidodendron grabaui* described here include vegetative organs of stems and leaves (Figure 3 and Figure 4), fertile structures (Figure 5 and Figure 6), and anatomy of the axis (Figure 7). Stems are up to 55 cm long and 5.5–33.5 mm wide (Figure 3A–G), displaying at least three dichotomies (Figure 3A–D). Each dichotomy produces two daughter axes of half-width, and the angles between the daughter axes are 30–60° (Figure 3A–F). 15.0–33.5 mm wide stems display elongated fusiform leaf bases (Figure 3G–L) that are up to 11.8 mm long and ca. 1.0 mm wide. All these leaf bases have a similar length/width ratio of ca. 12:1. Linear ornamentations occur on the interspaces between adjacent leaf bases (Figure 3G–J). Each leaf base displays a median fissure-like false leaf scar (i.e., the vertical “keel”), which occasionally shows an oval vascular bundle on the top (Figure 3K–M). Distal slender axes bear rhomboidal or fusiform leaf bases closely arranged in helices (Figure 4A–D). They are 3.4–5.5 mm long and 0.8–1.2 mm wide. These leaf bases have a similar length/width ratio (about 6:1), and each of them also bears a false leaf scar (Figure 4D). Vegetative leaves with entire margins are up to 12.4 mm long and inserted at 30–90° on the axis (Figure 4E–G). Each leaf has an obvious single vein extending from base to apex (Figure 4H,I).

The strobili are slender and cylindrical, 60–150 mm long and 6–11 mm wide, excluding sporophyll laminae (Figure 5A–J). Some strobili are fully preserved (Figure 5A), while the others are broken at distal or basal ends (Figure 5B–J). Most strobili are singly borne on the fertile axes (Figure 5B–D), while two specimens display paired strobili on dichotomy axes (Figure 5C,D), and two strobili are once-dichotomized (Figure 5E–G). The longest preserved strobilus is ca. 8 mm wide, broken at basal parts (Figure 5H). Three strobili are in the same direction (Figure 5I). Five strobili (arrows 1–5 in Figure 5J) are preserved in two directions (arrows 1–3 and arrows 4 and 5, respectively).

The central strobilar axes are up to 1.5 mm in width, bearing sporophylls in helices. The sporophylls are long-triangular in front view, ca. 2.0 mm at the widest part, showing a single vein in the middle (Figure 6A,B). Each sporophyll consists of a horizontal pedicel and an adaxially curved lamina (Figure 6C,E). All strobili were found to contain megaspores and are thus megasporangiate. Each sporophyll bears one sessile sporangium on the adaxial surface of the pedicel. However, only a few specimens show unbroken sporangial walls to recognize the contour of sporangia (Figure 6C–E). The sporangia are long-ellipsoidal in shape, 2.7–3.3 mm long, and 1.1–1.3 mm high (Figure 6D,E). One specimen shows two adjacent megaspore tetrads that are probably in one sporangium (Figure 6D). Isolated megasporophyll-sporangium complexes are ca. 3.2 mm long, showing exposed megaspores (Figure 6E). The megaspores are *Lagenicula*-type and 661–943 μm in diameter. The spores are round to pear-shaped in outline (Figure 6G–K). Each megaspore is composed of a spherical body and a distinct gula, with a gula height-to-body diameter ratio of 0.34 (Figure 6H,I). Spiny ornamentations, 18–77 μm long, are clear on some megaspores (Figure 6J,K).

The limonitized stele in Figure 3A (black arrow) is sliced and examined under the microscope. The primary xylem cylinder is exarch, ca. 3.8 mm in diameter (Figure 7A), without ridges of protoxylem. Protoxylem and metaxylem tracheids are circular or polygonal in transverse section, 20–30 μm and 40–120 μm in diameter, respectively (Figure 7B–D). One section displays a partially preserved metaxylem with torn tracheids (Figure 7C).

## 4. Comparisons

### 4.1. Comparison among Specimens of Sublepidodendron grabaui in China (Table 1)

*Sublepidodendron* [9,12,23,24,25] is cosmopolitan in the Late Devonian and Early Carboniferous. We consider our material represents the *Sublepidodendron grabaui* on the similarities of the following characteristics (Table 1): (1) correlation of horizon and age; (2) repeatedly bifurcated axes; (3) elongate fusiform leaf bases with evident fissure-shaped vertical false leaf scar (the length/width ratio of leaf bases, 6–12:1 in this study vs. 4–20: 1 in [23]); (4) wrinkles or longitudinal ornamentations between adjacent leaf bases; (5) paired slender strobili (60–150 mm × 6–11 mm in this study vs. 160 mm × 8.0 mm in [23]); (6) similar-shaped megasporophylls; (7) anatomy of the stele (solid exarch primary xylem). In this paper, we further provide the bifurcating nature of some *S. grabaui* megasporangiate strobili and illustrate the *Lagenicula* megaspores in them.

**Table 1:** Comparison among specimens of *Sublepidodendron grabaui* in China.

	*S. grabaui* (Wang and Xu [23])	*S. grabaui* (Meng et al. [12])	Specimens in this Study
**Vegetative axis**			
Width (mm)	1.5–100	3.0–43	3.0–33.5
Branching	at least four times-dichotomized	isotomously dichotomized or pseudomonopodially branched	at least thrice-dichotomized
Anatomy	The trunk: exarch primary xylem with a pith and secondary xylem; the branch: from the primary xylem with a pith to a solid primary xylem strand	A medullated stele or solid exarch primary xylem.	A solid exarch primary xylem.
**Leaf base**			
Shape	elongated fusiform	elongated fusiform	fusiform or elongated fusiform
Size (L × W, mm)	(1.6–20) × (0.3–1.2)	(3.0–8.5) × (0.5–1.3)	(3.4–12) × (0.8–1.2)
Long-width ratio	(4–20):1	7:1	(6–12):1
Vascular bundle scar	Present	–	Present
False leaf scar/“keel”	Present	Present	Present
**Strobilus**			
Shape	Cigar-shaped; singly or occasionally in pairs	Cigar-shaped	Cigar-shaped; singly, in pairs or occasionally once-dichotomized
Dimension (L × W, mm)	160 × 8.0	90 × (8.0–10)	(60–150) × (6–11)
Megasporangium	Elongate elliptical	Elliptical	Elongate ovoid
**Megaspore**			
Type	–	–	*Lagenicula*
Number per sporangium	–	–	8 or more
Diameter (μm)	1200	–	661–943 (gula/body = 0.34)
Ornamentation	–	–	spiny
**Microspore**			
Type	–	*Lycospora*	–
Diameter (μm)	–	18–35	–
Ornamentation	–	Finely microgranulate on surface	–

Note: –, lack of information.

### 4.2. Comparison with Lycopsids Bearing Bisporangiate Strobili (Table 2)

During the Middle and Late Devonian, lycopsids with bisporangiate strobili are widely distributed in the world (e.g., [5,20,21,22,26]). All these taxa display megasporophylls and microsporophylls in the basal and terminal parts of one strobilus (Table 2), respectively, and thus differ from *Sublepidodendron grabaui,* which possesses megasporangiate strobili.

**Table 2:** Comparison among *S. grabaui* and Devonian bisporangiate lycopsid representatives.

	*Sublepidodendron grabaui*	*Yuguangia ordinata*	*Kossoviella timanica*	*Cymastrobus irvingii*	*Clevelandodendron ohioensis*	*Kowieria alveoformis*
Reference	Wang and Xu [23]; Meng et al. [12]; this study	Hao et al. [5]	Orlova et al. [22]	Evreïnoff et al. [20]	Chitaley and Pigg [26]	Gess and Prestianni [21]
Locality	South China	South China	Russia	Australia	America	South Africa
Dimension of strobilus	60–160 mm long, 6–11 mm wide (L/W = 12–19)	up to 160 mm long, 8.6 mm wide (L/W = 18.6)	50–160 mm long, 2–8 mm wide (L/W = 20–25)	up to 80 mm long, 50 mm wide (L/W = 1.6)	90 mm long, 60 mm wide (L/W = 1.5)	up to 15 mm long
Attachement of terminal strobilus	singly, in pairs or occasionally once-dichotomized	singly or occasionally once-dichotomized	singly or occasionally once-dichotomized	singly	singly	singly
Megasporangium	Elongate ovoid	ovoid–elongate-ovoid	ovoid	oblate	oblate	ovoid
Megaspore diameter	661–943	450–832	450–1180	420–490	320–360	580–720
Megaspore number	8 or more	4 or more	8	multiple	multiple	Up to 4
Megaspore type	*Lagenicula*	*Triletes*	*Triletes*	*–*	*Triletes*	*Lagenicula*

Note: –, lack of information.

The Middle Devonian (Givetian) *Yuguangia ordinata* [5] and the Late Devonian (Frasnian) *Kossoviella timanica* [22] are bisporangiate lycopsids reported from South China and Northern Russia, respectively. These two plants bear slender and occasionally dichotomized strobili that are morphologically similar to *Sublepidodendron grabaui,* while they differ in the type of spores and shape of the sporangium.

*Cymastrobus irvingii* [20] and *Clevelandodendron ohioensis* [26] bear stubby strobili (length/width = 1.5–1.6) with oblate megasporangium. Each sporangium contains a large number of megaspores. *Clevelandodendron ohioensis* produces *Trileites*-type megaspores, while the megaspores of *Cymastrobus irvingii* show rows of papillae surrounding the trilete mark. In contrast, *Sublepidodendron grabaui* exhibits slender strobili (length/width = 11.7–13.8) and *Lagenicula*-type megaspores.

*Kowieria alveoformis* [21] from the Famennian of South Africa produces *Lagenicula* megaspores as in *Sublepidodendron grabaui*, while the numbers of megaspores per each sporangium in these two plants are different. Furthermore, *K. alveoformis* bears falcate sporophylls homomorphic to vegetative leaves that are dissimilar to those of *S. grabaui*.

### 4.3. Comparison with Lycopsids Bearing Monosporangiate Strobili from China (Table 3)

*Sublepidodendron songziense* occurs in the Upper Devonian Xiejingsi (previously known as Hsiehchingssu) Formation of the Hubei Province and the Wutong Formation of the Anhui Province, China. *S. songziense* and *S. grabaui* can be basically distinguished from each other by the different shapes of leaf bases and the ornamentations between them [24]. Detailed studies on these two plants’ *Lycospora*-type microspores show that the distal surface of the *S. songziense* microspore is granulated [24], while that of *S. grabaui* is microspinate [12]. Meanwhile, *S. songziense* is characterized by a complex branching system consisting of many lateral branches along its trunk, whereas *S. grabaui* probably displays bifurcations in stems but occasional pseudomonopodial branchings in slender axes (Table 3) and thus may exhibit a different architecture with *S. songziense.*

**Table 3:** Comparison among *S. grabaui* and related Devonian lycopsids bearing monosporangiate strobili from China.

	*Sublepidodendron grabaui*	*Sublepidodendron songziensis*	*Longostachys latisporophyllum*	*Minostrobus chaohuensis*	*Changxingia longifolia*	*Guangdedendron micrum*
Reference	Wang and Xu [22]; Meng et al. [12]; this study	Wang et al. [24,25]; Meng et al. [9]	Cai and Chen [6]	Meng et al. [8,11]	Wang et al. [10]	Wang et al. [27]; Gao et al. [14]
Branching system	at least thrice-dichotomized; isotomously dichotomized or pseudomonopodially branched	multiple dichotomized lateral branches	possible four times-dichotomized or more	eight times dichotomized at least	twice-dichotomized at least	twice-dichotomized at least
Leaf (length)	18–60 mm	10–15 mm	20–70 mm	5–7 mm	18–25 mm	20–92 mm
Leaf base /cushion (mm)	1.6–20 in length, 0.3–1.3 mm in width	1.0–3.5 in length, 0.8–1.5 in width	9.0–10.0 in length, 1.3–1.5 in width	6.0–9.0 in length, 1.0–1.6 in width	10.7–12.5 in length, 2.0–2.3 in width	15.6–22.7 in length, 2.6–4.3 in width
Shape of leaf base/cushion	fusiform or elongated fusiform	Fusiform, rhomboid or oval	narrow-fusiform	narrow-fusiform	rhomboid or fusiform	ovoid or narrow-fusiform
Anatomy	primary xylem with a pith and secondary xylem; a solid primary xylem strand	primary xylem, secondary xylem	primary xylem, secondary xylem	primary xylem	–	–
Megasporangia strobilus (mm)	60–160 in length, 6–11 in diameter; singly, in pairs or once-dichotomized	100–150 in length, 6.0–9.0 in diameter	30–225 in length, 7.0–10.0 in diameter; singly, occasionally in pairs	up to 125 in length, 4.5–6.0 in diameter	20–50 in length, 6.0–9.6 in diameter; singly, occasionally in pairs	50–234 in length, 9.0–24.0 in diameter; singly, in pairs or once-dichotomized
Sporangium	elongate ovoid	elongate-elliptical	round or ovoid	spherical to ellipsoidal	elliptical	long-ellipsoidal
Megaspore	661–943 (gula/body = 0.34)	gula/body = 0.51	1188–1425 μm in diameter	gula/body = 0.47	gula/body = 0.52	gula/body = 0.89
Megaspore number	8 or more	at least 20	4	4	probable 4	multiple
Megaspore type	*Lagenicula*	*Lagenicula*	*Laevigatisporites*?	*Lagenicula*	*Lagenicula*	*Lagenicula*

Note: –, lack of information.

*Longostachys latisporophyllus* [6] is a small arborescent lycopsid with megasporangiate strobili from the Middle Devonian (Givetian) of Hunan Province. Its stems are possibly at least four times dichotomized, and its leaves show spiny appendages. Slender strobili consist of spoon-shaped sporophylls and contain *Laevigatisporites*?-type megaspores. The megasporophyll of *Sublepidodendron grabaui* shows horizontal pedicel and upturned lamina and produces *Lagenicula* megaspores.

*Minostrobus chaohuensis* [8,11,28] is another arborescent lycopsid with monosporangiate strobili found in the Wutong formation from South China. Its aerial axes display multiple dichotomies and fusiform leaf bases/cushions. Protoxylem confined to ridges at the periphery of primary xylem. Each megasporangium of *M. chaohuensis* contains a single tetrad of *Lagenicula* megaspores. *Sublepidodendron grabaui* differs from *M. chaohuensis* in the number of megaspores in each sporangium and the shape of leaf bases/cushions.

*Changxingia longifolia* is also reported from the Upper Devonian (Famennian) of South China and is interpreted as a small-sized lycopsid with monosporangiate strobili [10,15]. Its rhomboidal leaf cushions and oblanceolate leaf bases are helically arranged on the stems. Both *C. longifolia* and *Sublepidodendron grabaui* produce dichotomized stem and terminal megasporangiate strobili in pairs, while the former bears shorter strobili (20–50 mm in length), and each sporangium contains four *Lagenicula*-type megaspores.

**Figure 8:**
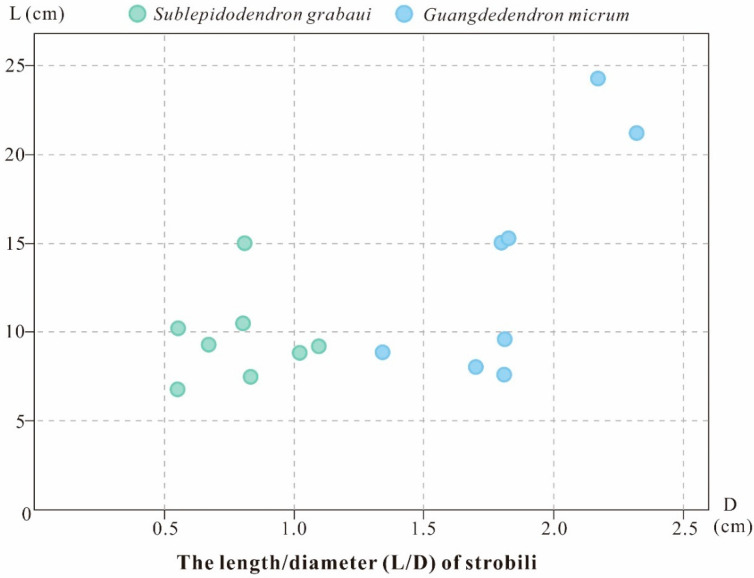
Comparison plot of the dimensions of *Sublepidodendron grabaui* and *Guangdedendron micrum* strobili. Data from Wang et al. [27], Gao et al. [14], and this study.

*Guangdedendron micrum* shares the same fossil locality with *Sublepidodendron grabaui* and is regarded as the major tree lycopsid that made up the Xinhang fossil forest [14,27]. In the Leigutai Member of Yongchuan Section, *G. micrum* is widely distributed, while *S. grabaui* is restricted to the bottom portion. In *G. micrum*, the strobili are borne singly, in pairs, and occasionally once-dichotomized, resembling some specimens of *S. grabaui*; however, the *S. grabaui* stems dichotomize several times, while *G. micrum* stems rarely branch. Furthermore, *S. grabaui* shows a much smaller overall architecture than *G. micrum*: the axes, strobili and leaves of the former are roughly half the size of those of the latter. The strobili of *G. micrum* are 70–240 mm in length and 13–23 mm in diameter, while those of *S. grabaui* are 60–150 mm and 6–11 mm (Figure 8). Along the thick stems or the main trunks, *G. micrum* displays a fusiform leaf cushion (the length/width ratio = 6:1) with an elliptical leaf scar in the middle, while *S. grabaui* shows slenderer, elongated fusiform leaf bases (the length/width ratio = 12:1) with a fissure-like false leaf scar. In addition, the *Lagenicula*-type megaspores of the two plants can be clearly distinguished: megaspores of *S. grabaui* show smaller gula (gula/body = 0.34), whereas those of *G. micrum* possess larger gula (gula/body = 0.89).

## 5. Discussion

Heterosporous lycopsids occurred in the Middle Devonian [4,5,6] and underwent their first evolutionary radiation during the Late Devonian [3,5]. The previous phylogenic result shows that heterosporous lycopsids with monosporangiate strobili originated in those with bisporangiate strobili and are proposed to be the most derived clade [29,30,31]. Wang et al. [32] considered that the reproductive diversification of arborescent lycopsids occurred in the Late Devonian according to *Lepidostrobus* specimens. Paired or bifurcated strobili occurred in Famennian *Sublepidodendron grabaui*, as well as *Guangdedendron micrum* and several other taxa, and such a trait was interpreted as a reproductive strategy to produce more sporangia [14].

*Lagenicula*-type megaspores are characterized by a prominent gula. Till now, almost all Late Devonian lycopsids with monosporangiate strobili found in South China produce *Lagenicula*-type megaspores (e.g., *Minostrobus chaohuensis, Sublepidodendron songziensis*, and *Guangdedendron micrum*). However, diversification also exists among these *Lagenicula* megaspores, i.e., the gula height-to-body diameter ratio of these Late Devonian lycopsids varies. Such ratio is 0.34–0.52 in *S. grabaui*, *M. chaohuensis*, *S. songziense* and *Changxingia longifolia*, while 0.89 in *G. micrum*. The varied dimensions of gula and body among different species could result in a difference in adaptability to the environment, as some evidence suggests that megaspores with a prominent gula could adapt to the dispersal from a highly dense canopy [33]. In this regard, *G. micrum* is better adapted to the high density of communities than *S. grabaui*, which is consistent with the quantitative difference we observed between them in the Xinhang Forest.

The reduced number of megaspores per megasporangium is considered as a derived trait in the Suborder Dichostrobiles of Order Isoëtales sensu lato [8]. The Late Devonian *Minostrobus* and *Changxingia* show four megaspores in each megasporangium, while the Carboniferous *Sigillaria* shows numerous ones [34] and may thus hint a mosaic pattern of characteristic evolution of heterosporous lycopsids [30]. Moreover, some other lycopsids, including *Kowieria* with bisporangiate strobili, and *Wuxia* with intercalary megasporangiate fertile zones, also contain four megaspores in each of their megasporangium [16,21]. Therefore, the reduced number of megaspores per megasporangium in lycopsids might be a trait with multiple independent origins beyond the Suborder Dichostrobiles.

## 6. Conclusions

We excavated new specimens of *Sublepidodendron grabaui* from Guangde City, Anhui Province, China, to further investigate its characteristics. This plant is now considered a tree lycopsid exhibiting multiply dichotomized stems and occasional lateral branches, and its terminal fertile axes may bear single, paired, or bifurcated megasporangiate strobili. Each megasporangium produces at least eight *Lagenicula* megaspores. Despite their relatively small quantity, *S. grabaui* was involved in the formation of the Xinhang Forest for some time.

## Data Availability

The specimen is deposited in the Department of Geology, Peking University, Beijing, China.
